# Validation of the Greek version of the distress thermometer compared to the clinical interview for depression

**DOI:** 10.1186/s12888-020-02926-0

**Published:** 2020-11-06

**Authors:** M. Kyranou, C. Varvara, M. Papathanasiou, Ι. Diakogiannis, K. Zafeiropoulos, M. Apostolidis, C. Papandreou, M. Syngelakis

**Affiliations:** 1 Department of Nursing, Cyprus University of Technology, Limassol, Cyprus; 2 Department of Medical Oncology, School of Medicine, Aristotle University of Thessaloniki, Papageorgiou Hospital, Thessaloniki, Greece; 3 Psycho-oncology Service, Division of Psychosomatic Medicine; First Psychiatric Clinic, School of Medicine, Aristotle University of Thessaloniki, Papageorgiou Hospital, Thessaloniki, Greece; 4 Department of International and European Studies, University of Macedonia, Thessaloniki, Greece

**Keywords:** Distress, Assessment, Oncology, Outpatient chemotherapy, Psychiatric interview

## Abstract

**Background:**

The Distress Thermometer (DT) is worldwide the most commonly used instrument for quick screening of emotional burden in patients with cancer. In order to validate the Greek version of the DT in the Greek population we aimed to explore the capacity of the DT to identify patients with comorbid depressive diagnosis.

**Methods:**

We analyzed the routinely collected clinical data from 152 patients with cancer who had been evaluated by the consultation-liaison psychiatric service and had received a diagnosis of either depressive disorder or no psychiatric diagnosis. The score of the DT accompanied by the list of problems in the Problem List, the depression status, and the clinical and demographic characteristics entered the data sheet.

**Results:**

The ROC analysis revealed that the DT achieved a significant discrimination with an area under the curve of 0.79. At a cut-off point of 4, the DT identified 85% of the patients with an ICD-10 depressive diagnosis (sensitivity) and 60% of the patients without a psychiatric diagnosis (specificity). The positive predictive value was 44%, the negative predictive value 92% and the diagnostic odd ratio 8.88. Fatigue and emotional difficulties were the most commonly reported problems by the patients.

**Conclusion:**

The Greek version of the DT has a sufficient overall accuracy in classifying patients regarding the existence of depressive disorders, in the oncology setting. Therefore, it can be considered as a valid *initial screening tool* for depression in patients with cancer; patients scoring ≥4 should be assessed by a more thorough mental evaluation.

## Background

According to the International Agency for Research on Cancer the global burden of cancer is estimated to have risen to 18.1 million new cases and 9.6 million deaths in 2018 [1]. In addition, the number of new cases is expected to grow from 18.1 million to 29.4 million by 2040 [1]. Thus, cancer becomes the primary disease leading to death in all countries during the twenty-firstcentury [2]. In Greece, the estimated number of new cases of patients with cancer during 2018 was 67,401, and the estimated number of deaths was 33,288, in a population of approximately 11 million people [2].

Cancer is associated with emotional burden. To describe it in a comprehensive and non-stigmatizing manner, the term *distress* has been introduced by the National Comprehensive Cancer Network® (NCCN®) defined as follows: “*Distress is a multifactorial unpleasant experience of a psychological (i.e., cognitive, behavioral, emotional), social, spiritual, and/or physical nature that may interfere with the ability to cope effectively with cancer, its physical symptoms, and its treatment. Distress extends along a continuum, ranging from common normal feelings of vulnerability, sadness, and fears to problems that can become disabling, such as depression, anxiety, panic, social isolation, and existential and spiritual crisis”* [3]. Distress is a universal phenomenon in patients with cancer; everyone at some point will experience some level of distress [4]. Roughly, a third of patients is estimated to experience high distress [5] although others suggest that the percentage of highly distressed patients is even larger [3, 6]. Most importantly, high distress interferes with patients’ decision making, and is associated with non-compliance and poor quality of life [3].

While many patients with cancer will affront mood fluctuations and a significant proportion will develop transient adjustment or mood disorders, around 10%, or more, will suffer from major depressive disorder (MDD) [7–9]; MDD can be considered as an extreme point on the distress continuum. The prevalence of MDD in patients with cancer is higher than this observed in the general population [7–9]. Of note, MDD is not the only nor the most prevalent depressive disorder in patients with cancer [9, 10]. Depression exacerbates anxiety, pain, and fatigue, reduces normal functioning and quality of life, and undermines adherence as well as the trusting relationship between the patient and the oncology team. It has also been hypothesized that depression is a significant negative predictor of survival [11, 12]. However, depression is both under-recognized and undertreated in the oncology setting [3, 7]. In a Scottish study of 21,151 cancer patients, out of 1538 patients who met the diagnostic criteria for clinical depression, 1130 (73%) did not receive any “potentially effective treatment” [7].

The pressing need to detect and reduce the emotional burden in patients with cancer can be partially addressed by the availability of screening tools. An accurate tool can help the oncologists expedite the diagnosis and can lessen the work-load of the limited psychosocial services. The Distress Thermometer (DT) is the most widely used instrument for quick screening of emotional burden in patients with cancer [13]. The DT is a visual analog scale (VAS) ranging from 0 to 10, resembling the format of the well-known Pain Thermometer. Usually, a score higher than 4 (or 5) is considered as *high distress* and is indicative of an increased probability for a diagnosis of a mental disorder necessitating a thorough psychosocial evaluation [9, 13]. There are currently 10 translations of the DT available through the NCCN® website (including the Greek translation) [14]. In a Greek study, the DT was compared to the Hospital Anxiety and Depression Scale (HADS) in elderly patients with colorectal cancer hospitalized in a surgical ward; the authors proposed 7 as the preferred cut-off point [15].

In this study, we aimed to explore the capacity of the Greek version of the DT to discriminate patients with cancer who suffer from clinical depression as defined by the International Classification of Diseases-10th edition (ICD-10).

## Methods

### Study participants

The participants were patients receiving chemotherapy for solid tumours at the Outpatient Clinic in the Department of Medical Oncology at Papageorgiou Hospital in Thessaloniki. Data from the evaluation of 152 patients with cancer in active treatment were used for this report.

### Procedure

We analyzed clinical data collected from patients who had been evaluated by the consultation-liaison (C-L) psychiatric service. Written consent was obtained from the participants. Participants consist of patients who had received a psychiatric diagnosis of either depressive disorder or no psychiatric disorder. Interviews were conducted by a resident and two skilled C-L nurses supervised by two licensed psychiatrists. A structured interview (the Mini International Neuropsychiatric Interview, MINI) was used and the diagnosis was reached as a result of a unanimous clinical decision of the C-L team. Patients were also asked by the nurses of the Oncology Department to complete the Distress Thermometer. The clinicians who made the psychiatric diagnosis where blinded to the results of the distress thermometer.

### Measures

Demographic and clinical information was obtained by the medical records.

#### Distress thermometer

The distress thermometer is a single item questionnaire presented by Roth et al. [16]. It is based on a numeric scale ranging from 0 to 10, where 0 indicates no distress and 10 indicates extreme distress. The DT is accompanied by a comprehensive list of problems faced by patients, known as “Problem List”, in which common problems/symptoms are presented in five dimensions: practical problems, family problems, emotional problems, spiritual/religious concerns and physical problems.

With the approval and collaboration of the NCCN, the Greek edition of the Distress Thermometer [17] was developed. We used forward-translation and back-translation process and the draft was sent to the NCCN team for review and verification. The translation team of the NCCN requested some revisions which were all addressed reaching the final version of the Greek edition of the Distress Thermometer [translated by Kyranou, Varvara, Syngelakis, Dec 2014].

#### Psychiatric clinical interview

MINI is a structured psychiatric evaluation for diagnosing mental disorders according to the DSM-IV/ICD-10 criteria. MINI is very convenient in clinical/research settings since it can be completed in less than 30 min, and has sufficient psychometric properties [18, 19].

### Data analysis

The Statistical Package for Social Science (SPSS), version 25, for Mac was used for the analysis of the data. Descriptive statistics were used for the calculation of frequencies of the demographic characteristics and the problems faced by patients.

Receiver operating characteristic (ROC) analysis was used to define the DT cut off score in order to discriminate the depressed from the non-depressed patients. To identify the best cut-off point we used the Younden index, the empirical criterion of estimating the maximum distance from the diagonal reference line, and the diagnostic odd ratio (DOR) criterion. Sensitivity, specificity, positive predictive value (PPV) and negative predictive value (NPV) were also calculated.

In order to discriminate between groups of patients and their characteristics and to examine the correlation between groups of patients, chi-square tests were used. Independent t-tests were performed to compare between groups of patients and the number of problems they reported on the Problem List, and between the characteristics of the participants and the number of problems they reported.

## Results

### Descriptive statistics

The characteristics of the participants are presented in Table 1. The mean score of the DT in our sample was 3.60; SD: 3.15.

**Table 1:** Characteristics of the participants [*N* = 152]

	N	%
Age	mean:58.9 SD:12.1	
Gender		
Male	67	44.0
Female	85	56.0
Diagnosis Ca		
Breast	21	13.8
Gastrointestinal	49	32.2
Gynaecological	35	23.0
Lung	14	9.2
Head & Neck	14	9.2
Place of Living		
Thessaloniki	56	36.8
Other city	30	19.7
Urban area	47	30.9
Family status		
Single	16	10.5
Married	94	61.8
Widow	13	8.6
Divorced	12	7.9
Occupation		
Housekeeping	22	14.5
Unemployment	21	13.8
Retirement	61	40.1
Education		
Primary	53	34.9
Secondary	45	29.6
University	35	23.0

### ROC analysis

**Fig. 1:**
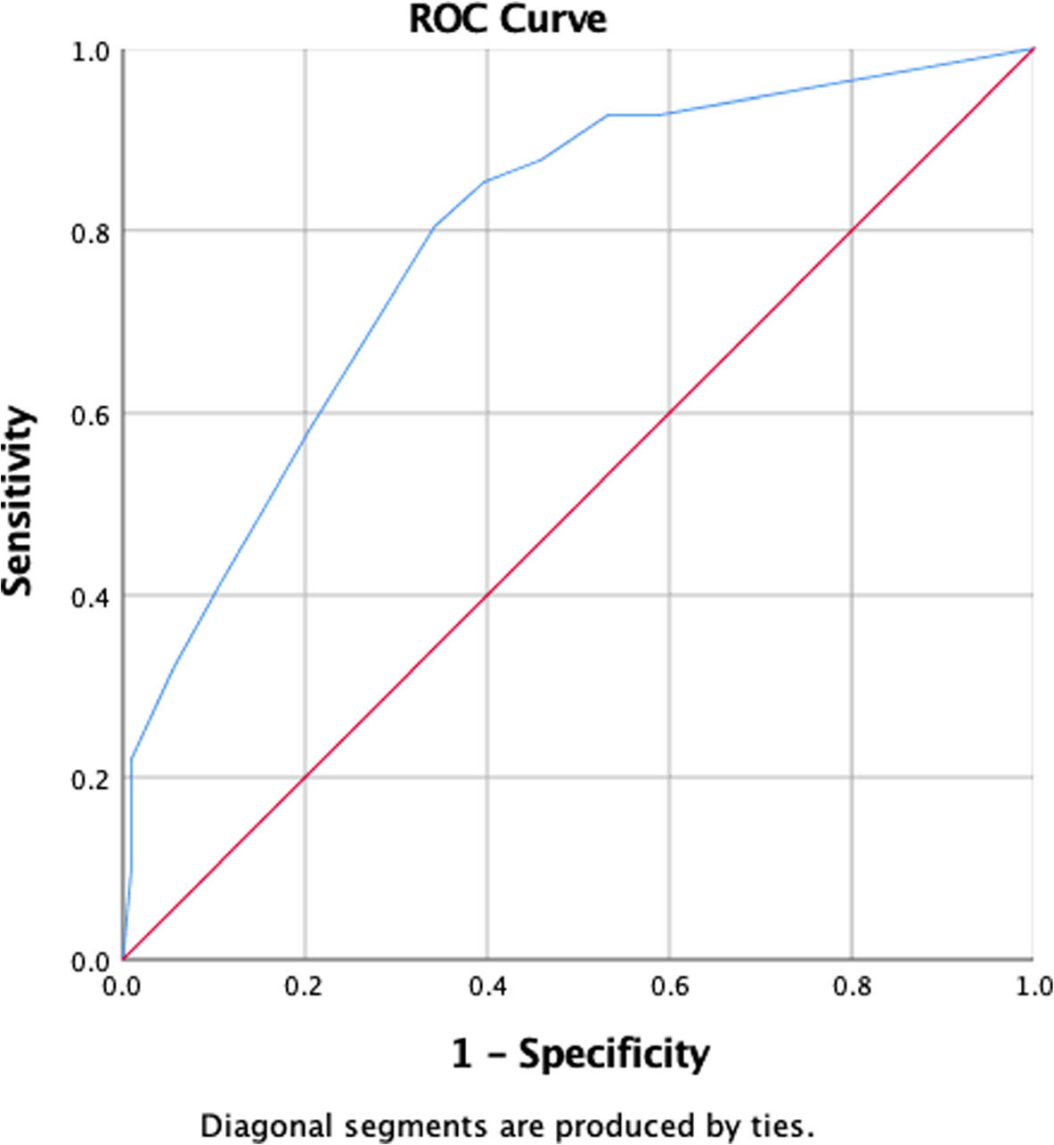
The AUC comparing the DT with the Clinical Interview

The ROC analyses showed that the DT had a fair/good discrimination capacity compared to the clinical interview with an area under the curve (AUC) of **0.79** [SE = 0.04, 95% (0.71, 0.87), *p* < 0.01] as shown in Fig. 1.

The Youden index and the empirical criterion for locating the maximum distance from the diagonal reference line are presented in Table 2. The point 5 (Youden Index = 0.461, Maximum Distance = 0.326) seems to be the best balanced cut-off point; the results for a cut-off point of 5 are slightly better than those corresponding to a cut-off point of 4 (Youden Index = 0.456, Maximum Distance = 0.322).

**Table 2:** Younden’s Index and Maximum Distance

Cut off point	Sensitivity	Specificity	Youden Index
0	1	0	0
0.5	0.93	0.41	0.341
1.5	0.93	0.47	0.395
2.5	0.88	0.54	0.419
3.5	0.85	0.60	0.458
***4***	***0.85***	***0.60***	***0.456***
4.5	0.81	0.66	0.463
***5***	***0.80***	***0.66***	***0.461***
5.5	0.59	0.79	0.378
6.5	0.42	0.89	0.307
7.5	0.32	0.95	0.263
8.5	0.22	0.99	0.211
9.5	0.10	0.99	0.089
10	0	1	0

On the contrary, the cut-off point of 4 functioned better than the cut-off point of 5 when the DOR was applied for the estimation of the discriminatory capacity (DOR _for 4_ = 8.883, DOR _for 5_ = 7.924). In this frame, sensitivity was increased up to 0.85 at the expense of a lower specificity (Table 3). The importance of the DT from a clinical point of view is that it can identify as many patients with clinical depression as possible; in this case also the choice of the point 4 is the most appropriate cut-off point.

**Table 3:** Percentage of correct classification for a cut off score of 4 & 5

DT distressed cut-off point	ICD-10 depressed TP/FN	non-depressed FP/TN	Sens	Spec	PPV	NPV	YI	Max Dist.	DOR
≥4	35/6	44/67	**0.85**	0,60	0,44	0,92	0.456	0.322	**8.883**
≥5	33/8	38/73	0.80	0.66	0,46	0,90	**0.461**	**0.326**	7.924

*DT* Distress Thermometer, *ICD-10* International Classification of Diseases, 10th revision, *TP* True Positive, *FN* False Negative, *FP* False Positive, *TN* True Negative, *Sens* Sensitivity, *Spec* Specificity, *PPV* Positive Predictive Value, *NPV* Negative Predictive Value, *YI* Youden Index, *Max Dist* Maximum Distance from the diagonal reference line, *DOR* Diagnostic Odds Ratio

### Sociodemographic characteristics of groups of patients

The relationship between distressed / non-distressed patients and their gender was significant [x^2^(1, 152) =8.032, *p* ≤ .005] women scored higher than men. The groups did not differ in any of the other patients’ sociodemographic characteristic (age, place of living, family status, occupation, education). There was no significant relationship between depressed/non-depressed patients and any sociodemographic characteristic.

### Problems reported in the problem list

Problems reported in the Problem List are presented in Table 4.

**Table 4:** Problems reported in the Problem List

	Distressed DT > 4		Non-Distressed		*p*	Depressed		Non-Depressed		*p*
	n	%	n	%		n	%	n	%	
Practical Problems										
Child care	11	13.9	4	5.5		7	17.1	8	7.2	
Housing	14	17.7	5	6.8	*	6	14.6	13	11.7	
Insurance/Financial	25	31.6	11	15.1	*	14	34.1	22	19.8	
Transportation	15	19.0	13	17.8		7	17.1	21	18.9	
Work	12	15.2	4	5.5		5	12.2	11	9.9	
Treatment decisions	26	32.9	9	12.3	**	10	24.4	25	22.5	
Family Problems										
Dealing with children	12	15.2	7	9.6		6	14.6	13	11.7	
Dealing with partner	16	20.3	5	6.8	*	5	12.2	16	14.4	
Ability to have children	3	3.8	6	8.2		2	4.9	7	6.3	
Family health issues	24	30.4	7	9.6	**	15	36.6	16	14.4	**
Emotional Problems										
Depression	21	26.6	2	2.7	**	15	36.6	8	7.2	**
Fears	41	51.9	10	13.7	**	22	53.7	29	26.1	**
Nervousness	38	48.1	16	21.9	**	23	56.1	31	27.9	**
Sadness	42	53.2	7	9.6	**	23	56.1	26	23.4	**
Worry	53	67.1	22	30.1	**	27	65.9	48	43.2	*
Loss of interest in usual activities	34	43.0	6	8.2	**	18	43.9	22	19.8	**
Spiritual/Religious Concerns	11	13.9	7	9.6		6	14.6	12	10.8	
Physical Problems										
Appearance	22	27.8	10	13.7	*	12	29.3	20	18.0	
Bathing/dressing	26	32.9	13	17.8	*	13	31.7	26	23.4	
Breathing	16	20.3	4	5.5	**	9	22.0	11	9.9	
Changes in urination	18	22.8	9	12.3		12	29.3	15	13.5	*
Constipation	31	39.2	16	21.9	*	17	41.5	30	27.0	
Diarrhea	23	29.1	9	12.3	**	8	19.5	24	21.6	
Eating	31	39.2	11	15.1	**	20	48.8	22	19.8	**
Fatigue	58	73.4	25	34.2	**	30	73.2	53	47.7	**
Feeling swollen	22	27.8	10	13.7	*	12	29.3	20	18.0	
Fevers	10	12.7	2	2.7	*	3	7.3	9	8.1	
Getting around	25	31.6	7	9.6	**	14	34.1	18	16.2	*
Indigestion	14	17.7	7	9.6		11	26.8	10	9.0	**
Memory/concentration	19	24.1	9	12.3		14	34.1	14	12.6	**
Mouth sores	8	10.1	5	6.8		6	14.6	7	6.3	
Nausea	33	41.8	7	9.6	**	15	36.6	25	22.5	
Nose dry/congested	21	26.6	11	15.1	*	9	22.0	23	20.7	
Pain	36	45.6	6	8.2	**	15	36.6	27	24.3	
Sexual	11	13.9	13	17.8		2	4.9	22	19.8	*
Skin dry/itchy	23	29.1	10	12.3	*	15	36.6	18	16.2	**
Sleep	27	34.2	11	15.1	**	15	36.6	23	20.7	
Substance abuse	2	2.5	1	1.4		1	2.4	2	1.8	
Tingling in hands/feet	36	45.6	21	28.8	*	21	51.2	36	32.4	*

* *p* < 0.05 **p ≤ 0.01

## Discussion

In a convenient sample of patients with cancer, with mixed diagnosis, receiving chemotherapy in an outpatient clinic, the Greek version of the DT compared to the clinical psychiatric interview demonstrated sufficient accuracy in classifying patients with depressive disorders.

The AUC was 0.79. Searching for the optimal cut-off point we faced a dilemma since 4 and 5 had similar operating characteristics. At a cut-off point of ≥4 the sensitivity was 0.85, the specificity 0.60, the PPV 0.44, the NPV 0.92 and the DOR 8.88. At a cut-off point of ≥5 the sensitivity was 0.81, the specificity 0.66, the DOR 7.92 whereas the Youden Index was slightly higher. We decided to choose 4 as the proposed cut-off point because from a clinical point of view at this cut-off point the test performs better (higher DOR, sensitivity exceeding the 0.85 level).

Our decision is not only clinically relevant but also recommended. Ma et al. [13] in their meta-analysis faced the same dilemma in the comparison of the DT to the DSM-IV. From their part, they chose a higher DOR and a higher sensitivity instead of a slightly better Youden Index. Thus, they recommended 4 as the optimal cut-off score “in order to rule in as many cases”. Furthermore, consistency on a global scale was an additional important criterion for adopting 4 as the cut-off score in the Greek Version as 4 is the preferred cut-off point worldwide [13].

The psychometric properties of the DT have been examined during the last 20 years, compared to several different tools. Paradoxically, in examining a screening test which of course attempts to detect firstly the most severely distressed people, i.e. those with a psychiatric diagnosis, the gold standard, the clinical interview, has not been commonly utilized. Ma et al. [13] in their meta-analysis examining the accuracy of the DT included 42 eligible studies from 20 counties in which 10 different reference standards were used. Only 8 of the 42 studies used “the real standard (the clinical interview)” while the others used questionnaires, mainly the HADS; researchers consider this finding as a limitation in their meta-analysis. Accordingly, Donovan et al. [20], in their research for translated versions of the DT, presented 23 publications describing the use of a non-validated foreign language version of the DT. Only in four of them mental diagnosis, following clinical interview, was utilized as a criterion in the ROC analysis.

In our study, DT *showed a good sensitivity* of 85% but a relatively low specificity of 60%. According to Ma et al. [13], when all the results were pooled together the DT, at the cut-off point of 4, demonstrated “a good balance between pooled sensitivity (0.81, 95% CI 0.79-0.82) and pooled specificity (0.72, 95% CI 0.71-0.72)”. When DT was compared to HADS-Total “the balance between pooled sensitivity (0.82, 95% CI 0.80-0.84) and pooled specificity (0.73, 95% CI 0.72-0.74) was maximized”. At the same cut-off point, in the comparison of the DT to the clinical interview/DSM-IV, the pooled sensitivity was 0.84 (95% CI 0.80–0.88) but the pooled specificity dropped to 0.63 (95% CI 0.61–0.66). Finally, in the comparison of the DT to the clinical interview/ICD-10 the pooled sensitivity was 0.79 (95% CI 0.60–0.87) and the pooled specificity 0.60 (95% CI 0.52–0.68). It is worth mentioning that in a previously published meta-analysis the psychometric properties were found even lower [21], while there are some studies that failed to find a link between the DT and the clinical interview [22, 23].

Few studies have focused in the ability of the DT to identify depressive disorders compared to the clinical interview. Akizuki et al. [24] reported that DT revealed a sensitivity of 84% and specificity of 61% for detection of adjustment disorders and major depression. Grassi et al. [25] found a sensitivity of 79.5% and specificity of 75.4%, following an ICD-10 diagnosis of affective syndrome. Rooney et al. [26] reported a sensitivity of 94 to 67% (at different time points) and specificity of 69 to 75% for MDD; researchers investigated the operating characteristics of HADS, PHQ-9 and DT and they concluded that “due to a modest positive predictive value of either instrument, patients scoring above these thresholds need a clinical assessment to diagnose or exclude depression”. On the other hand, in the Wagner et al. study [27] – where DT, Hopkins Symptom Check List-25 (HSCL-25), PHQ-9/PHQ-2 and Structured Clinical Interview (SCID) for major depression, dysthymia, and adjustment disorders were used – the DT showed a sensitivity of 0.80% and a specificity of 52%; the authors underlined that: “The NCCN®-DT (AUC=0.59) indicated poor accuracy in classifying patients with regard to the presence of mood disorders.”

Our results are in agreement with those derived by most researchers who used the psychiatric interview as the gold standard to validate the DT’s accuracy; the Greek version of the NCCN®‘s Distress Thermometer exhibited at least similar psychometric properties to previous reports from other international studies. Additionally, our results support a 2-step process; patients scoring ≥4 should undergo a more thorough mental evaluation.

The psychometric properties of the DT have raised a debate regarding its usefulness. Recklitis et al. [28] in their study of the DT compared to a psychiatric interview reported a sensitivity of 68.18%, and a specificity of 78.33%; they emphasized that “The DT … failed to identify 31.81% of survivors with a SCID diagnosis. No alternative DT cut-off score met criteria for acceptable sensitivity (≥.85) and specificity (≥.75).” Wagner et al. [27] extend similar concerns to an extreme by questioning the DT as useless.

Given the necessity of detecting mental problems in patients with cancer, various instruments are offered to clinicians to assist them identify patients in need for psychosocial support. Oncologists seem to face difficulties in recognizing the psychiatric morbidity [22, 29]. The nurses are often the first point of encounter with the patient and as such can be extremely assisted by a brief measure of psychological distress screening [30]. The DT belongs in the category of Ultra Short Term Questionnaires; in the clinical setting these tests are very easy to administer, quick and inexpensive. Nevertheless, their feasibility is counterbalanced by a modest accuracy and a poor specificity [21]. It would be worth noting that short tests do operate better when applied to rule out non-depressed patients [9, 31] In a busy oncology department, it would be extremely useful for the clinicians to be aware of the patients not suffering from depression. As for those highly distressed, a more thorough assessment of a possible diagnosis of depression can be utilized [9, 26, 31, 32].

As expected distressed/depressed patients reported more problems on the Problem List compared with non-distressed/non-depressed patients. The most frequent problems reported by the distressed/depressed patients were fatigue, followed by emotional problems, more specifically worries and nervousness; while pain and sleep were reported at a high percentage, spiritual/religious concerns, child care and sexual problems were in contrary at a low percentage. ‘Sexual problems’ was the only item in which more non-depressed than depressed patients expressed concerns to a significant point. However, the lack of randomization cannot exclude the possibility of selection bias in our sample.

In a previous Greek study, Antoniadis et al. [15] compared the DT with the HADS in elderly (mean age: 70, SD: 9.5) patients with colorectal cancer who were admitted for surgery in a period of surgical treatment; the researchers excluded patients with major health problems as well as those with a psychiatric history during the past 5 years. “Compared to cancer patients from other countries the mean HADS score of [their] sample was significantly higher” [15]. The mean score of DT was 5.7 (sd 2.7), the AUC 0.805 and for the cut-off point of 7, sensitivity was 0.73, specificity 0.80. In the Problem List worries (81.0%), nervousness (78.6%), fears (70.2%), treatment decisions (69.0%), sleep (67.9%), sadness (65.5%), child care (59.5%) and fatigue (52.4%) were the most reported. According to the authors, cultural factors may have contributed to the differences, especially for the high cut-off score; they also speculated that the socioeconomic condition in Greece and the economic crisis may have had an impact. Our results are not in agreement with these assumptions. Greek cultural factors or socioeconomic condition did not differentiate our results, which are similar to those reported from other countries [13]. Possibly, the sampling procedure and the treatment phase had a crucial influence on the differences reported by Antoniadis et al. Of note, DT scores may differ at different time points on the cancer trajectory [26, 33].

Several limitations to this study need to be acknowledged. This was a single-center study, at a University Hospital with patients in active treatment. We used a non-random sample and the numbers do not allow for comparisons between patients suffering from different types of cancer or being on different chemotherapy regimens. Finally, we did not search for possible subtypes within the construct of depression. A multi-center study, with a large heterogeneous sample will allow for more detailed comparisons between subgroups of patients on different points within the illness trajectory.

## Conclusions

The Greek version of the Distress Thermometer performs well, at the cut-off point of 4, in classifying patients regarding the existence of depressive disorders; it can be utilized in a 2-step approach to diagnose MDD or related disorders. We consider the validation of the Greek version of the DT, a well-known international screening tool, as our contribution to lessen the emotional burden of our patients, and as a step forward in the underserved area of psychosocial interventions.

## Data Availability

The dataset used and analysed during the current study are available on reasonable request.

## Abbreviations

AUC: Area Under the Curve
DOR: Diagnostic Odd Ratio
DSM: Diagnostic and Statistical Manual of Mental Disorders
DT: Distress Thermometer
HADS: Hospital Anxiety and Depression Scale
ICD-10: International Classification of Diseases-10th edition
MDD: Major Depressive Disorder
MINI: Mini International Neuropsychiatric Interview
NCCN®: National Comprehensive Cancer Network®
NPV: Negative Predictive Value
PHQ: Patient Health Questionnaire
PPV: Positive Predictive Value
ROC: Receiver Operating Characteristic
SCID: Structured Clinical Interview for DSM
SPSS: Statistical Package for Social Science
VAS: Visual Analog Scale
